# Removal of an osteointegrated broken uncemented femoral stem after hip arthroplasty—technical note

**DOI:** 10.1186/s13018-021-02365-x

**Published:** 2021-03-26

**Authors:** Peter Wahl, Theo Solinger, Michel Schläppi, Emanuel Gautier

**Affiliations:** 1 Department of Orthopaedics, HFR Fribourg – Cantonal Hospital, Fribourg, Switzerland; 2 Division of Orthopaedics and Traumatology, Cantonal Hospital Winterthur, Brauerstrasse 15, P.O. box 834, 8401 Winterthur, Switzerland; 3 Orthopaedic Clinic Rosenberg, St. Gallen, Switzerland

**Keywords:** Hip arthroplasty, Revision, Broken stem, Removal, Extraction, Transfemoral

## Abstract

Broken stems are particularly challenging in revision hip arthroplasty, as no standard extraction instruments fit anymore. Well-integrated uncemented stem remnants can be particularly arduous to remove. Stem fatigue failure is not rare with modular stems. Since these are particularly useful in revision hip arthroplasty, increasing numbers of broken stems are to be expected. Usually applied techniques using cortical fenestration distally to the tip of the stem or using an extended transfemoral approach cause supplementary bone defects impairing reconstruction. We present a relatively simple and reproducible revision technique, using a limited standard approach and only regular orthopedic instruments, to extract the remnants of broken uncemented femoral stems in hip arthroplasty. This technique was applied successfully and without complications in 6 cases, permitting eventually the reimplantation of even shorter stems.

## Introduction

Fracture of the femoral stem accounts for approximately 1% of revisions after primary total hip arthroplasty (THA) [1–3]. Following revision THA (rTHA), the prevalence may even be higher, making out up to 2–3% of subsequent revisions [3, 4]. Modular stems have a much higher associated fracture rate [5]. While modularity offers advantages over monobloc stems regarding reliability of the reconstruction and consequently functional results in rTHA [6–8], worrisome incidences of stem fractures have been reported, at least for certain models [5, 6, 9–11]. Electrocautery-induced damage on the neck, at either primary implantation or following stem-retaining revision, may be a another particular cause of stem fracture [12, 13].

However, removal of a broken, distally well-fixed stem can be challenging, as no standard extraction device can be adapted anymore to the distal element [14]. Reported technical solutions include extensive operative approaches or the application of devices used off-label or custom-made. A commonly used technique is to access the full length of the stem through a long transfemoral approach (extended trochanteric osteotomy) [14–16], but this requires anchoring the new stem further distally or the use of devices allowing fixation with locking bolts. Another option is a distal fenestration for retrograde removal of the implant, but this causes distal bone defects and stress risers [14, 17]. Alternatives are to use custom-made instruments, or a supplementary approach through the knee, requiring off-label use of an intramedullary nail for retrograde removal [18–20].

**Fig. 1:**
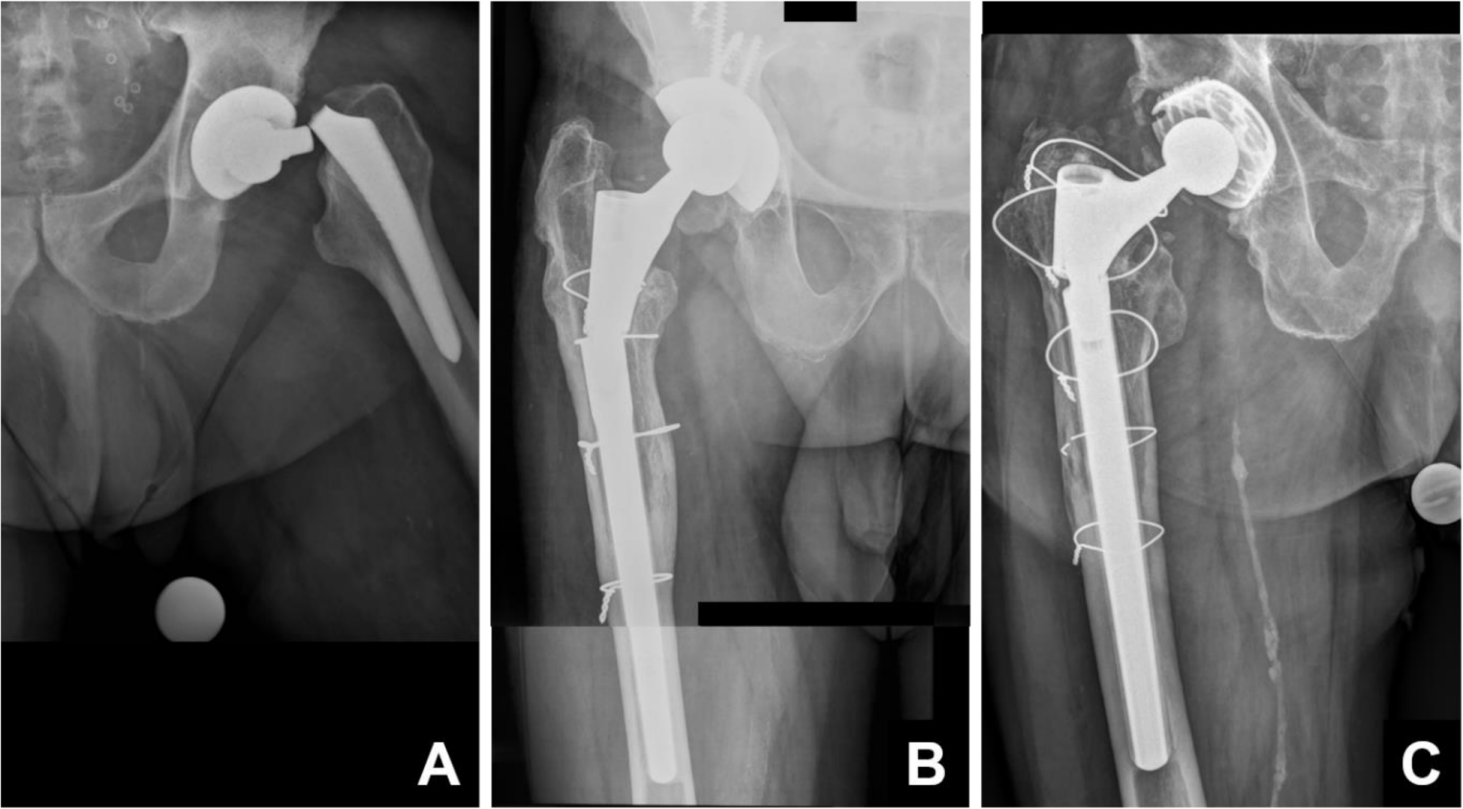
Zone of interest of the anteroposterior pelvic radiographs of three different patients presenting with a well-integrated, broken, uncemented stem after hip arthroplasty. All cases have in common that no standard extraction component can be fitted anymore. In **a**, a 76-year-old male patient presenting less than 4 years after stem-preserving revision with exchange of the head, performed due to instability, with a well-fixed uncemented primary stem (AMIStem-H, Medacta, Castel San Pietro, Switzerland) broken at the neck. Analysis of the retrievals confirmed electrocautery damage as the cause of the fatigue fracture. In **b**, a 72-year-old male patient presenting 6 years after stem exchange performed due to a periprosthetic fracture through a transfemoral approach, with reconstruction performed with a tapered, fluted, modular, straight 200-mm stem with distal anchoring (Revitan straight, Zimmer Biomet, Winterthur, Switzerland). Failure was at the level of the connection pin, which had milled out from the bore within the distal component of the stem. In **c**, a 73-year-old male patient presenting with recurrent failure of the modular connection of a stem implanted 15 years earlier through a transfemoral approach. The proximal component is obviously angulated and loosened again. Reconstruction had been performed with a tapered, fluted, modular, straight 200-mm stem with distal anchoring (PFM-R, Zimmer Biomet, Winterthur, Switzerland) due to aseptic loosening of a previously implanted cemented stem. Nine years later, another revision with exchange of the proximal component due to loosening of the modular connection had been performed

We present a relatively simple technique for extraction of broken femoral stem remnants in rTHA, using a limited standard exposure and standard instruments. This method has now been used successfully in 6 cases, with either a broken primary stem or broken modular revision stems (Fig. 1).

## Surgical technique

**Fig. 2:**
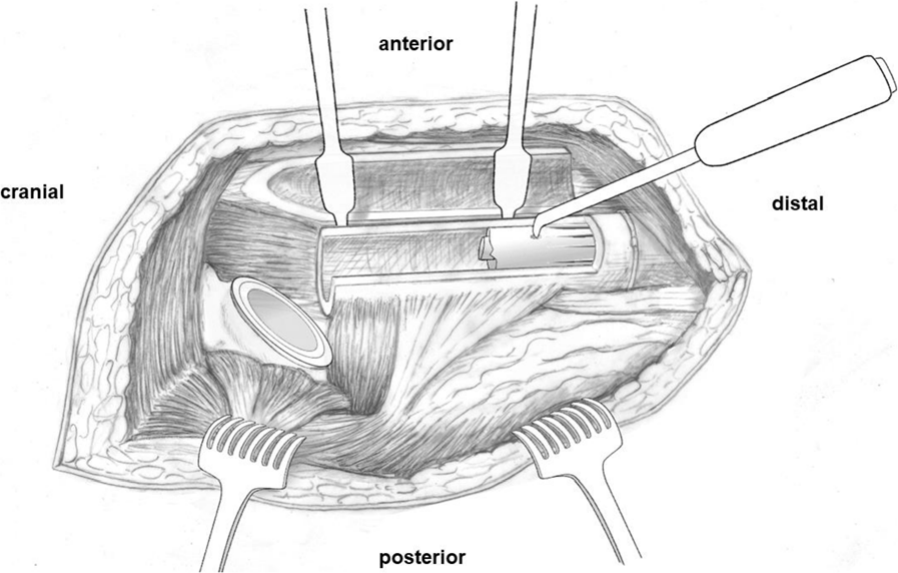
Drawing of the fully exposed transfemoral approach to the right hip. The fragment of the extended trochanteric osteotomy is reclined anteriorly, behind the Hohmann retractors. The proximal component of the broken, distally well-integrated, uncemented stem already has been removed. A tapered, fluted, uncemented stem is exemplified. The angulated pointed stem extractor is in place in the recess made in the stem with a carbide drill bit. The distal part of the stem might have to be loosened by longitudinal drilling along the stem, before it can be struck out

Revision is performed in a lateral position through a transfemoral approach (extended trochanteric osteotomy) [15, 16]. The osteotomy is performed between 120 and 170 mm distally to the tip of the greater trochanter. The osteotomy thus offers exposure to the proximal extremity of the well-fixed distal fragment of the stem (Fig. 2). For broken revision stems, exposing 30 to 40 mm of the stem distally to the level of the fracture is enough. The stem chosen for reconstruction also has to be considered when planning, in order to ensure good distal stem purchase with a connection zone of any modular stem proximal to the osteotomy, as it often has a larger diameter than the medullary cavity.

A cerclage should be in place around the intact diaphysis just distally to the osteotomy, to avoid fractures during extraction as well as during impaction of the new stem [15]. A recess was drilled into the proximal side of the stem, using a 6.5-mm carbide drill (HSS, DePuy Synthes, Zuchwil, Switzerland). This allowed anchoring an angulated pointed stem extractor (Ref. 75.85.75, Zimmer Biomet, Winterthur, Switzerland) to strike out the component (Fig. 2). In case of a long, solidly integrated segment, partial loosening of the stem might first have to be achieved by longitudinal drilling along the implant, using long flexible 2.5-mm drill bits (Ref. 315.920, DePuy Synthes).

## Discussion

Fatigue failure of the femoral stem is a known issue in hip arthroplasty [1–4]. Modularity is known to be associated with a major increase in the incidence of this problem [5]. Modularity however is one of the key elements of successful stem revision as it allows separating anchoring the stem from reconstruction of leg length and torsion [7]. Patient-related outcomes consequently are better with modular stems than with monobloc stems [6–8]. Increasing use of modular revision stems will invariably lead to increasing numbers of broken stems [5, 6, 9–11]. Another issue may be fracture of stems due to fatigue failure following electrosurgery-induced damage at the level of the neck, either at primary implantation or following stem-retaining revision [12, 13]. Every surgeon involved in rTHA should be aware of the problem and familiar with appropriate extraction and reconstruction techniques [14].

Many surgeons involved in rTHA know the proposed technique. However, to the best of our knowledge, it had not been described appropriately so far. The authors do not pretend to any first application. The technique described does not require special or custom-made instruments. It also does not require extending the approach to the distal femur, nor cortical fenestration of the distal femur, nor a supplementary transgenicular approach [14, 17–20]. To drill a recess into the side of the stem remnant, we recommend using a large diameter carbide drill bit. Initiation with a smaller diameter drill bit might be necessary, particularly with stems not made of titanium alloy. Carbide has a hardness of 9–9.5 on the Mohs scale and is therefore much harder than the titanium alloys used for uncemented stems, which are around 6 on the Mohs scale. The harder stainless steel alloys of some stems, being around 8 on the Mohs scale, would still be softer than carbide and thus accessible to the same technique.

Longitudinal drilling might be useful to loosen partially and to ease striking out well-integrated components. For this, we recommend using long 2.5-mm drill bits as these are slightly flexible, reducing the risk of breakage. Fluted stems are particularly well suited for this, as the flutes guide the drill bit. Threaded k-wires may be used too, but are not recommended as they cause strain, which might lead to fractures or heat-induced necrosis of the bone. Chisels might also cause fractures, due to the thickness of the blade, and perforations might be more difficult to handle than those from a relatively small drill bit. Drilling had to be performed in two of the five tapered, fluted stems.

No fracture was caused during the extraction procedure in all 6 cases. Reconstruction was performed successfully in all cases using an uncemented, tapered, fluted, modular revision stem.

## Data Availability

Not applicable.

## Abbreviations

rTHA: Revision total hip arthroplasty
THA: Total hip arthroplasty
